# Hypoxanthine-guanine phosphoribosyltransferase and inosine 5′-monophosphate dehydrogenase activities in three mammalian species: aquatic (*Mirounga angustirostris*), semi-aquatic (*Lontra longicaudis annectens*) and terrestrial (*Sus scrofa*)

**DOI:** 10.3389/fphys.2015.00212

**Published:** 2015-07-29

**Authors:** Myrna Barjau Pérez-Milicua, Tania Zenteno-Savín, Daniel E. Crocker, Juan P. Gallo-Reynoso

**Affiliations:** ^1^Programa de Planeación Ambiental y Conservación, Laboratorio de Estrés Oxidativo, Centro de Investigaciones Biológicas del NoroesteLa Paz, Mexico; ^2^Department of Biology, Sonoma State UniversityRohnert Park, CA, USA; ^3^Laboratorio de Ecofisiología, Centro de Investigación en Alimentación y DesarrolloGuaymas, Mexico

**Keywords:** hypoxia, hypoxanthine-guanine phosphorybosiltransferase, inosine 5′-monophosphate dehydrogenase, purine salvage, purine synthesis

## Abstract

Aquatic and semiaquatic mammals have the capacity of breath hold (apnea) diving. Northern elephant seals (*Mirounga angustirostris*) have the ability to perform deep and long duration dives; during a routine dive, adults can hold their breath for 25 min. Neotropical river otters (*Lontra longicaudis annectens*) can hold their breath for about 30 s. Such periods of apnea may result in reduced oxygen concentration (hypoxia) and reduced blood supply (ischemia) to tissues. Production of adenosine 5′-triphosphate (ATP) requires oxygen, and most mammalian species, like the domestic pig (*Sus scrofa*), are not adapted to tolerate hypoxia and ischemia, conditions that result in ATP degradation. The objective of this study was to explore the differences in purine synthesis and recycling in erythrocytes and plasma of three mammalian species adapted to different environments: aquatic (northern elephant seal) (*n* = 11), semiaquatic (neotropical river otter) (*n* = 4), and terrestrial (domestic pig) (*n* = 11). Enzymatic activity of hypoxanthine-guanine phosphoribosyltransferase (HGPRT) was determined by spectrophotometry, and activity of inosine 5′-monophosphate dehydrogenase (IMPDH) and the concentration of hypoxanthine (HX), inosine 5′-monophosphate (IMP), adenosine 5′-monophosphate (AMP), adenosine 5′-diphosphate (ADP), ATP, guanosine 5′-diphosphate (GDP), guanosine 5′-triphosphate (GTP), and xanthosine 5′-monophosphate (XMP) were determined by high-performance liquid chromatography (HPLC). The activities of HGPRT and IMPDH and the concentration of HX, IMP, AMP, ADP, ATP, GTP, and XMP in erythrocytes of domestic pigs were higher than in erythrocytes of northern elephant seals and river otters. These results suggest that under basal conditions (no diving, sleep apnea or exercise), aquatic, and semiaquatic mammals have less purine mobilization than their terrestrial counterparts.

## Introduction

Purine nucleotides and their related metabolic products are involved in many biological processes (Carver, 1999; Pang et al., 2012). One of the most important functions of purine nucleotides, such as adenosine 5′-triphosphate (ATP), is to provide energy required by all living cells for the maintenance of metabolism (Janssen, 1993; Hochachka and Somero, 2002). The majority of ATP production is oxygen (O_2_)-dependent (Storey, 1996). Most mammalian species have low or no tolerance to lack of oxygen, and cells and tissues become compromised during prolonged episodes of decreased tissue oxygen content (hypoxia) (Boutilier and St-Pierre, 2000). However, aquatic mammals such as elephant seals and sperm whales can tolerate prolonged periods of hypoxia, related to dive-associated breath hold (apneas) (Elsner, 1999). Breath-hold diving is accompanied by decreased heart rate (bradycardia) and peripheral vasoconstriction, which reduces blood flow (ischemia) to peripheral organs that are tolerant to hypoxia (skin, muscle, kidney, liver) and deflects the oxygenated blood to the brain and nervous system, which are sensitive to hypoxic conditions (Elsner, 1999; Castellini and Castellini, 2004; Panneton, 2013). Northern elephant seals (*Mirounga angustirostris*) dive continuously while at sea, to mean depths over 500 m and mean durations over 23 min, followed by short surface intervals of 2–3 min (Robinson et al., 2012). These dives are associated with unusual degrees of systemic hypoxia (Meir et al., 2009). Semiaquatic mammals, such as river otters, also have the ability to dive. Their routine dives last approximately 30 s, with a maximum reported depth of 14 m; however, the majority (98%) of their dives occur in shallow waters, less than 8 m deep (Nolet et al., 1993; Bertonatti and Parera, 1994; Kruuk, 1995). Although terrestrial mammals, such as humans and domestic pigs, are not adapted to diving, the diving response (bradycardia, peripheral vasoconstriction, blood flow redistribution) is similar to that observed in aquatic and semiaquatic mammals; the extent of the response (reduction of heart rate, for instance) is greater in diving mammals (Panneton, 2013).

Despite the adaptations to diving and the physiological adjustments during diving, when oxygen reserves are depleted, blood and tissues become hypoxic and ATP hydrolysis results in the accumulation of xanthine and uric acid, purine metabolites which can not be recycled (Janssen, 1993; Elsner, 1999). An alternative mechanism for maintenance of the purine nucleotide pool is the salvage pathway, which conserves energy and uses preformed bases from degradation of nucleic acids and from the diet (Alexiou and Leese, 1992; Carver, 1999; Moriwaki et al., 1999; Nyhan, 2005; Zhang et al., 2008). Nucleotides such as inosine 5′-monophosphate (IMP), guanosine 5′-monophosphate (GMP), and adenosine 5′- monophosphate (AMP) can be regenerated by this pathway (Carver, 1999; Zhang et al., 2008). The enzyme hypoxanthine-guanine phosphoribosyltranferase (HGPRT, E.C. 2.4.2.8) produces IMP and GMP from 5′-phosphoribosyl-1-pyrophosphate (PRPP) and hypoxanthine (HX) and guanine, respectively (Stout and Caskey, 1985; Craig and Eakin, 2000). IMP is used by the enzyme inosine 5′-monophosphate dehydrogenase (IMPDH, E.C. 1.1.1.205) to produce xanthosine 5′-monophosphate (XMP), is an intermediate metabolite for the synthesis of guanine nucleotides (Sintchak and Nimmesgern, 2000).

HX accumulation was observed in the kidney and heart of domestic pigs, but not in the same tissues of ringed seals, after experimental ischemia (Elsner et al., 1998). HX accumulation in plasma from young weaned northern elephant seals was reported after apneas related to both sleep and voluntary dives (Vázquez-Medina et al., 2011). Increased HGPRT activity, as well as HX accumulation, were reported for northern elephant seal pups at different stages of the post-weaning fasting (Soñanez-Organis et al., 2012).

The hypothesis of this study was that aquatic mammals, such as the northern elephant seal, have a higher capacity for purine recycling than semi-aquatic or terrestrial mammals. Therefore, the goal of this study was to analyze HGPRT and IMPDH activities, as well as the concentration of their related purine metabolites (HX, IMP, AMP, ADP, ATP, GDP, GTP, and XMP) in erythrocytes and plasma from three species of mammals adapted to different environments, aquatic (northern elephant seal), semiaquatic (neotropical river otter), and terrestrial (domestic pig), under normal conditions (no diving, sleep apnea or exercise).

## Materials and methods

### Samples

All samples were collected from healthy individuals. Blood samples from northern elephant seals were collected under NMFS marine mammal permit # 14636. All procedures were approved by Sonoma State University IACUC. Blood samples were collected from northern elephant seals (*M. angustirostris*, *n* = 11; 4 males, 7 females; on average 126.3 ± 13.5 kg body mass; approximately 0.8 years old), river otters (*L. longicaudis annectens*, *n* = 4; 2 males, 2 females, 6.84 ± 0.8 kg body mass; 3.3 ± 1.4 years old), and domestic pigs (*S. scrofa*, *n* = 11; sex and weight not determined). Northern elephant seal samples were collected at Año Nuevo Reserve, CA, USA in collaboration with University of California, Merced and Sonoma State University. Blood samples were obtained from sedated animals as previously reported (Ortiz et al., 2003; Vázquez-Medina et al., 2011). River otter samples were obtained from captive animals at Acuario de Veracruz, México. Blood sampling was part of routine procedures to check the otter's health status; animals were sedated as described previously (López-Cruz et al., 2014). Samples from domestic pigs were collected post-mortem at the local slaughterhouse in La Paz, Baja California Sur. Blood was drawn from the jugular vein in a period no longer than 30 min after sacrifice. All blood samples were collected under asceptic conditions in Vacutainer® tubes containing ethylenediaminetetraacetic acid (EDTA) as anticoagulant. To recover plasma, samples were centrifuged on site in a field centrifuge (Mobilespin 126 Vulcon Technologies, Grandview, MO, USA) at 850 × g for 10 min. Buffy coat was discarded, red blood cells were washed using cold saline solution (0.9%). All samples were kept on ice during transportation to the laboratory and stored at −80°C until analyzed. Prior to each analysis, intraerythrocyte content was obtained by osmotic shock with cold distilled water, followed by two cycles of freezing/thawing and centrifuged at 9000 × g for 15 min.

### Biochemical analyses

#### Enzyme activity

Hypoxanthine-guanine phosphorybosiltransferase (HGPRT, E.C. 2.4.2.8) activity was quantified in erythrocytes and plasma samples using the PRECICE HPRT® Assay Kit (NOVOCIB, Lyon, France) following the manufacturer's instructions. Briefly, samples were incubated at 37°C with a mixture of dithiothreitol (DTT), NAD^+^, IMPDH, reaction buffer, and PRPP. In this assay, HGPRT activity is measured as the rate of production of IMP, which is oxidized by IMPDH. Change in absorbance at 340 nm was recorded every 5 min for 120 min using a microplate reader (Multiskan FC, Thermo Scientific, Finland). HGPRT activity was expressed as nmol mg^−1^ protein h^−1^. HGPRT activity detection limit was 0.004 nmol mg^−1^ protein h^−1^.

Inosine 5′-monophosphate dehydrogenase (IMPDH, E.C. 1.1.1.205) activity was determined by quantifying the concentration of xanthosine 5′-monophosphate (XMP), resulting from the conversion of IMP, using high performance liquid chromatography (HPLC) (Waters 2695, Milford, MA, USA) (Glander et al., 2001). In each run, a standard curve of XMP was performed at a concentration range from 0.39 to 50 μM mL^−1^. Only in this technique, the erythrocytes were lysed by diluting the sample in a cold solution of distilled water and DTT (4 mM) and, previous to the analysis, were incubated for 3 h at 37°C, in a mixture of sodium phosphate buffer (40 mM), IMP (0.5 mM), NAD^+^ (0.25 mM), and KCl (50 mM) at pH 7.4. To stop the reaction, cold perchloric acid (HClO_4_, 4 M) was added; the reaction mixture was neutralized with potassium carbonate (KCO_3_, 5 M) and incubated at −80°C for 30 min. The extract of interest was obtained by filtering the neutralized reaction mixture through a Millex GV membrane of 0.22 mm (Int. Millipore, Bedford, MA, USA), and was placed in an HPLC vial and analyzed in duplicate. An ODS Hypersil 125 × 4.6 mm, 3 μm particle size column (Thermo Scientific, USA) was used as a stationary phase, while the eluent phase consisted of a binary gradient begining at 100% buffer A (KH_2_PO_4_ 0.1 M and tetrabutylammonium, TBA 8 mM, pH 6.0) to 100% buffer B (KH_2_PO_4_ 0.1 M, TBA 8 mM and methanol 30% v/v, pH 6.0). XMP detection in the samples was conducted based on the wavelength and the retention time of the peak of interest from each sample. XMP concentration (μM mL^−1^) was calculated from the standard curve and the area of the corresponding peak in the XMP chromatogram. One unit of IMPDH is defined as the amount of enzyme needed to produce 1 mM of XMP. IMPDH activity was expressed as nmol mg^−1^ protein h^−1^. IMPDH activity detection limit was 4.28 U mg^−1^ protein h^−1^.

#### Purine metabolite concentration

Concentration of purines (HX, IMP, AMP, ADP, ATP, XMP, GDP, and GTP) was quantified in erythrocytes and plasma samples by HPLC (Waters 2695, Milford, MA, USA) (Gianattassio et al., 2003). To quantify the concentration of each purine metabolite, a curve with a standard mixture of known concentrations (0.39–50 μM mL^−1^) was used. Samples were treated with cold perchloric acid (HClO_4_, 0.5 M) to remove all except the compounds of interest; the mixture was neutralized with potassium hydroxide (KOH, 0.5 M) and monobasic potassium phosphate (KH_2_PO_4_, 1 M, pH 7.5). The extract of interest was obtained by filtering the mixture through a Millex GV membrane of 0.22 mm (Int. Millipore, Bedford, MA, USA), and it was placed in an HPLC vial and analyzed in duplicate. A Supelcosil LC-18, 150 × 4.6 mm, 3 μm particle size column was used as a stationary phase. The eluent phase consisted of potassium phosphate buffer solution (0.1 M), TBA (8 mM), pH 6.0 with and without acetonitrile (CH_3_CN, 30% v/v) at room temperature. Concentration of each purine metabolite in erythrocytes and plasma samples was calculated from the standard curve and the area of the corresponding peaks in the chromatogram. Purine metabolite concentrations were expressed as μM mg^−1^ protein.

#### Total soluble protein

In order to standardize results, total soluble proteins were quantified using a commercial kit (Bio-Rad Laboratories, Inc. Hercules, CA, USA) based on Bradford (1976), using bovine serum albumin (BSA) as the standard, and a microplate reader (Multiskan FC, Thermo Scientific, Finland). Total protein content in erythrocytes and plasma samples was calculated from the BSA standard curve (0.005–0.2 mg mL^−1^) and results were expressed in mg protein mL^−1^.

### Statistical analyses

Data were grouped by species; normality and homocedasticity of the data were tested using Kolmogorov–Smirnoff (*p*>0.05) and Levene (*p* < 0.05) tests, respectively. Given the non-normal distribution of the data, significant differences were determined with Kruskall–Wallis and Mann–Whitney's *U*-tests (Zar, 1999). Statistical significance was considered when *p*≤0.05. Statistical analyses were performed using SYSTAT©v12.0 (SPSS, Inc. Chicago, IL, USA).

## Results

### Enzyme activity

**Figure 1:**
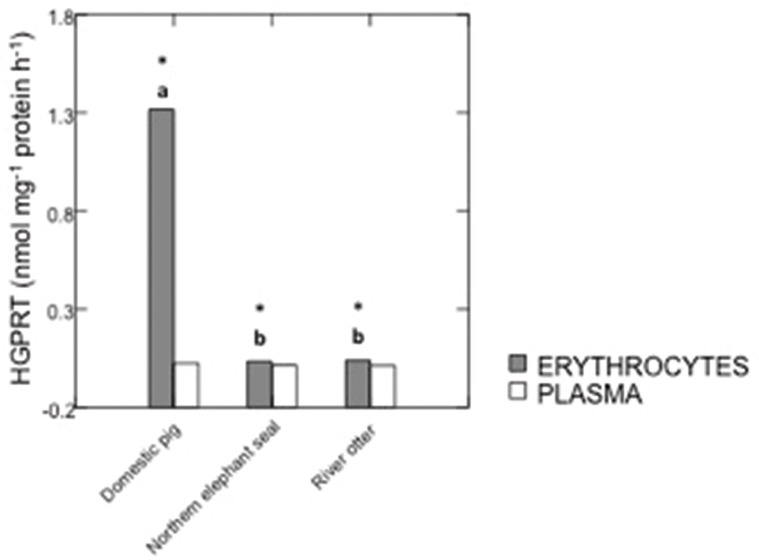
**Activity of hypoxanthine-guanine phosphorybosiltransferase (HGPRT, nmol mg^−1^ protein h^−1^) in erythrocytes and plasma from domestic pigs (***S. scrofa***, ***n*** = 11), river otters (***L***. ***longicaudis annectens***, ***n*** = 4), and northern elephant seals (***M. angustirostris***, ***n*** = 11)**. Data are presented as median. ^*^Significant differences between erythrocytes and plasma for each species, *p* ≤ *0.05*. Different letters denote significant differences between species, *p* ≤ *0.05*.

**Figure 2:**
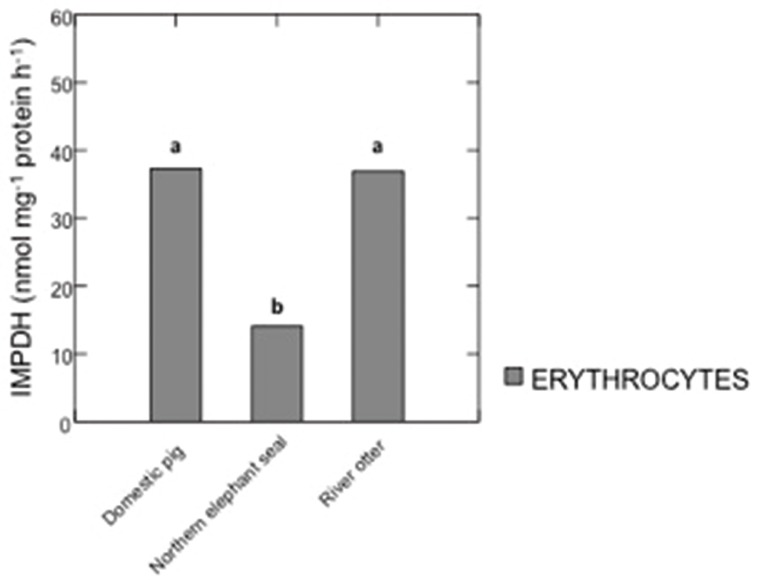
**Activity of inosine 5′-monophosphate dehydrogenase (IMPDH, nmol mg^−1^ protein h^−1^) in erythrocytes from domestic pigs (***S. scrofa***, ***n*** = 11), river otters (***L.longicaudis annectens***, ***n*** = 4), and northern elephant seals (***M. angustirostris***, ***n*** = 11)**. Data are presented as median. Different letters denote significant differences between species, *p* ≤ 0.05. IMPDH activity in plasma samples from domestic pigs, river otters, and northern elephant seals was below detection limit.

HGPRT and IMPDH activities in samples from domestic pigs, river otters, and northern elephant seals are summarized in Figures 1, 2. IMPDH activity in plasma samples from domestic pigs, river otters, and northern elephant seals was below detection limit. HGPRT activity was significantly higher in erythrocytes than plasma of domestic pig (*p* < 0.001), river otter (*p* = 0.021), and northern elephant seal (*p* = 0.017). HGPRT activity was significantly higher in erythrocytes of domestic pigs than in those from river otters (*p* < 0.001) and northern elephant seals (*p* < 0.001). However, HGPRT activity in erythrocytes from river otters was not significantly different from those in erythrocytes from northern elephant seals. HGPRT activity in plasma samples from river otters and northern elephant seals was marginally lower (NS, *p* = 0.09) than in those from domestic pigs. IMPDH activity was significantly lower in erythrocytes from northern elephant seals than in those from river otters and domestic pigs (*p* = 0.011). IMPDH activity in erythrocytes from river otters was not significantly different from those in domestic pigs.

### Purine metabolite concentration

HX, IMP, AMP, ADP, ATP, XMP, GDT, and GTP concentrations in erythrocytes and plasma samples from domestic pigs, river otters, and northern elephant seals are summarized in Table 1. HX concentration in erythrocytes from northern elephant seals and in plasma from river otters and northern elephant seals was below detection limit. HX concentration was lower in erythrocytes than in plasma from domestic pigs (*p* = 0.002). HX concentration in erythrocytes was higher in domestic pigs than in river otters (*p* = 0.004). IMP concentration in erythrocytes from northern elephant seals, as well as that in plasma of all three species analyzed was below detection limit. IMP concentration in erythrocytes from domestic pigs was significantly higher than in those from river otters (*p* = 0.004). AMP concentration in plasma of river otters and northern elephant seals was below detection limit. AMP concentration in domestic pigs was significantly higher in erythrocytes than in plasma (*p* < 0.001). AMP concentration in erythrocytes from domestic pigs was higher than in river otters pigs and northern elephant seals (*p* < 0.001); there were no significant differences between the latter. ADP concentration in plasma from river otters was below detection limit. ADP concentration in domestic pigs and northern elephant seals was significantly higher in erythrocytes than in plasma (*p* = 0.037). ADP concentration in erythrocytes was higher in domestic pigs than in river otters and in northern elephant seals (*p* < 0.001), but was not significantly different between river otters and northern elephant seals. ATP concentration in plasma from river otters and northern elephant seals was below detection limit. ATP concentration in domestic pigs was higher in erythrocytes than in plasma (*p* < 0.05). ATP concentration in erythrocytes was higher (NS, *p*>0.05) in domestic pigs than in river otters and northern elephant seals. GDP concentration in domestic pig was higher in erythrocytes than in plasma (*p* = 0.005). GDP concentration in erythrocytes from domestic pigs was significantly higher than in those from river otters (*p* = 0.037). GTP concentration in plasma from domestic pigs was below detection limit. GTP concentration in erythrocytes was significantly higher than in plasma for both river otters and northern elephant seals (*p* < 0.05). GTP concentration in erythrocytes was significantly higher in domestic pigs than in those from river otters and northern elephant seals (*p* < 0.05); there were no significant differences between river otters and northern elephant seals. XMP concentrations in plasma was below detection limit for all three species analyzed. In erythrocytes, XMP concentration was significantly lower in northern elephant seals than in domestic pigs and river otters (*p* < 0.05); there were no significant differences between the latter.

Table 1**Concentration (μM mg^−1^ protein) of hypoxanthine (HX), inosine 5′-monophosphate (IMP), adenosine 5′-monophosphate (AMP), adenosine 5′-diphosphate (ADP), adenosine 5′-triphosphate (ATP), guanosine 5′-diphosphate (GDP), guanosine 5′-triphosphate (GTP), and xanthosine 5′-monophosphate in erythrocytes and plasma from domestic pig (***S. scrofa***, ***n*** = 11), river otter (***L. longicaudis annectens***, ***n*** = 4), and northern elephant seal (***M. angustirostris***, ***n*** = 11)**.

**Table:** 

**Median (25th–75th percentiles)**										
	**HX (μM mg**^**−1**^ **protein)**		**IMP (μM mg**^**−1**^ **protein)**		**AMP (μM mg**^**−1**^ **protein)**		**ADP (μM mg**^**−1**^ **protein)**		**ATP (μM mg**^**−1**^ **protein)**	
	**Erythrocytes**	**Plasma**	**Erythrocytes**	**Plasma**	**Erythrocytes**	**Plasma**	**Erythrocytes**	**Plasma**	**Erythrocytes**	**Plasma**
Domestic pig	0.362 (0.282, 0.429)	1.463^*^ (0.715, 2.047)	1.034^A^ (0.925, 1.567)	ND	2.088^*^^A^ (1.957, 2.736)	0.002 (0.002, 0.016)	1.344^*^^A^ (0.806, 1.615)	0.010 (0.002, 0.016)	0.818^*^ (0.296, 1.059)	0.002 (0.002, 0.009)
River otter	0.113 (0.081, 0.136)	ND	0.100^B^ (0.056, 0.112)	ND	0.258^B^ (0.167, 0.301)	ND	0.406^B^ (0.276, 0.534)	ND	0.682 (0.537, 0.847)	ND
Northern elephant seal	ND	ND	ND	ND	0.131^B^ (0.114, 0.172)	ND	0.329^*^^B^ (0.302, 0.373)	0.012 (0.011, 0.013)	0.679 (0.560, 0.808)	ND

**Table:** 

**Median (25th–75th percentiles)**						
	**GDP (μM mg**^−1^ **protein)**		**GTP (μM mg**^−1^ **protein)**		**XMP (μM mg**^−1^ **protein)**	
	**Erythrocytes**	**Plasma**	**Erythrocytes**	**Plasma**	**Erythrocytes**	**Plasma**
Domestic pig	0.025^*^^A^ (0.023, 0.215)	0.002 (0.002, 0.021)	0.175^A^ (0.130, 0.220)	ND	0.112^A^ (0.096, 0.143)	ND
River otter	0.012^B^ (0.011, 0.038)	ND	0.072^*^^B^ (0.059, 0.076)	0.001 (0.001, 0.004)	0.111^A^ (0.067, 0.121)	ND
Northern elephant seal	ND	ND	0.046^*^^B^ (0.044, 0.055)	0.002 (0.002, 0.010)	0.042^B^ (0.027, 0.048)	ND

*ND = below detection limit*.

* *Significant differences between erythrocytes and plasma for each species, p ≤ 0.05. Different letters denote significant differences between species, p ≤ 0.05*.

## Discussion

HGPRT activity was significantly higher in erythrocytes and plasma from a non-diving mammal (domestic pig) than from diving mammals (river otter and northern elephant seal). Ninety percent of free purines (such as HX and guanine) are salvaged in humans purine metabolism (Stout and Caskey, 1985). HGPRT activity in human erythrocytes has been reported to be 69–96 U mg^−1^ protein (Elder and Johnson, 1983). It has been suggested that a higher HGPRT activity in human erythrocytes is associated with an efficient purine recycling capacity. Therefore, it is possible that, under normal conditions (no diving, sleep apnea, or exercise), erythrocytes from terrestrial mammals have a greater purine salvage capacity via HGPRT than erythrocytes from aquatic and semiaquatic mammals. Perhaps, if samples were to be collected during diving or exercise, the activity of HGPRT in aquatic mammals would be higher than that in terrestrial mammals—as happens during fasting and hypoxia. Furthermore, HGPRT activity in plasma of northern elephant seal underyearlings (this study) is lower than that reported in pups of 10–11 weeks of age, suggesting an increase in purine recycling as an adaptive response to prolonged fasting (a condition that increases nucleotide degradation) (Soñanez-Organis et al., 2012) and/or diving capacity. In comparison, this age class has spent several months at sea foraging, where extreme tissue hypoxia is a routine aspect of breath-holds (Meir et al., 2009).

IMPDH activity in erythrocytes from domestic pig was found to be higher than that of erythrocytes from river otter and northern elephant seal (this study). To our knowledge, there are no reports of IMPDH activity in these species. However, IMPDH activity has been reported in human erythrocytes from healthy individuals (85 pmol mg^−1^ protein h^−1^) and also in individuals with purine metabolic disorders (HGPRT deficiency, 234 pmol mg^−1^ protein h^−1^) (Montero et al., 1995). In either case, IMPDH activity was lower than that reported in this study. In the case of northern elephant seals, a lower IMPDH activity under normal conditions is probably due to the developmental capacity to supress their metabolism during breath-holds, which has been reported ~40% below their resting metabolic rate, and starts to develop during the post-weaning fasting period (Tift et al., 2013). While pups and underyearlings might not have the same oxygen storage capacity as that of adults, they exhibit deep and long-duration dives on their first trip to sea (Thorson and Le Boeuf, 1994), and these could impact different aspects of the energy metabolism (such as purine synthesis) during their time on land after returning from their first foraging trip.

Purine nucleosides (IMP and XMP) and nucleotides (AMP, ADP, ATP, GDP, GTP) concentrations in plasma from terrestrial mammals (human, rabbit and rat) are generally lower than their intracellular (erythrocytes, placenta, liver, heart) concentrations (Traut, 1994). This is consistent with the concentrations of purine metabolites found in plasma and erythrocytes of all three species analyzed in this study. Lower concentrations of purine bases in extracellular fluids may reflect an active uptake of these compounds into cells (Traut, 1994). The concentrations of purine metabolites depend on the unique functions of each cell type (erythrocytes, lymphocytes, liver) and even on the developmental phase of the cells (Traut, 1994). In erythrocytes from humans and rats, purine nucleotides cannot be synthesized *de novo*, and can only be reconstructed from pre-existing free purine bases or nucleosides (Baranowska-Bosiacka and Hlynczak, 2004), which can contribute to explain the lower concentrations of HX and higher concentrations of purine nucleosides and nucleotides in erythrocytes of all three species analyzed in this study. The higher concentration of HX in heart and kidney of domestic pigs, as compared to ringed seals, after experimental ischemia, suggests an increase of ATP degradation in domestic pigs (Elsner et al., 1998). This is consistent with the higher HX concentration found in erythrocytes and plasma of domestic pigs as compared to that from northern elephant seals and river otters (this study), and suggest an adaptation to avoid ATP degradation and accumulation of purine nucleotides in response to diving in marine mammals. It is possible that differences in dietary nucleotide content may affect the concentations of metabolites and activity of enzymes involved in purine synthesis and degradation leading to the differences observed in this study between aquatic, semi-aquatic and terrestrial mammals; further studies to explore this are warranted.

In summary, HGPRT and IMPDH activities as well as HX, IMP, XMP and guanine (GDP and GTP), and adenine (AMP, ADP and ATP) nucleotides concentrations in plasma and erythrocytes from diving mammals were lower than those from a non-diving mammal. These results suggest that terrestrial mammals rely on both purine *de novo* synthesis and recycling, while diving mammals primarily utilize the *de novo* pathway to produce guanine nucleotides under normal conditions (no diving, sleep apnea, or exercise). Aquatic mammals have anatomical, biochemical, and physiological adaptations that allow them to tolerate periods of apnea, hypoxia, and ischemia associated to diving (Reviewed in Kooyman et al., 1981; Elsner, 1999; Castellini and Castellini, 2004; Costa, 2007; Panneton, 2013). These may include a less active purine salvage pathway in diving mammals, which could increase during diving, sleep apnea, exercise and/or fasting (conditions that are known to enhance ATP degradation in non-diving mammals) as has been previously suggested (Elsner et al., 1998; Soñanez-Organis et al., 2012). Further studies are required to elucidate the contribution of the *de novo* and salvage pathways in purine metabolism in aquatic, semiaquatic, and terrestrial mammals in response to diving, sleep apnea, exercise, and fasting.

## Author contributions

MB, TZ, DC, and JG contributed by drafting this manuscript and revising it critically. MB contributed with sample analysis. MB and TZ analyzed data. MB, TZ, and DC help with interpretation of data. DC provided northern elephant seal samples.

## Conflict of interest statement

The authors declare that the research was conducted in the absence of any commercial or financial relationships that could be construed as a potential conflict of interest.
